# Tear Sampling and Biomarker Discovery: A Robust Workflow for Routine Clinical Applications Using UHPLC-MS/MS and Schirmer Strips

**DOI:** 10.3390/ijms26052041

**Published:** 2025-02-26

**Authors:** Rossana Comito, Carmen Ciavarella, Gloria Astolfi, Matteo Conti, Emanuele Porru, Francesco Saverio Violante, Piera Versura

**Affiliations:** 1 Division of Occupational Medicine, IRCCS Azienda Ospedaliero-Universitaria di Bologna, 40138 Bologna, Italy; rossana.comito@aosp.bo.it (R.C.); francesco.violante@unibo.it (F.S.V.); 2 Ophthalmology Unit, DIMEC, Alma Mater Studiorum Università di Bologna, 40138 Bologna, Italy; carmen.ciavarella2@unibo.it (C.C.); gloria.astolfi2@unibo.it (G.A.); piera.versura@unibo.it (P.V.); 3 Department of Public Health, Health Service of the Emilia-Romagna Region, 40026 Imola, Italy; matteo.conti@ausl.imola.bo.it; 4 Biostructures and Biosystems National Institute “INBB”, 00136 Rome, Italy; 5 Occupational Medicine Unit, Department of Medical and Surgical Sciences, Alma Mater Studiorum, University of Bologna, 40138 Bologna, Italy; 6 IRCCS Azienda Ospedaliero-Universitaria di Bologna, 40138 Bologna, Italy

**Keywords:** tear film, proteomics, Schirmer strip, mass spectrometry, non-invasive matrices, cancer biomarkers

## Abstract

Human tear analysis is gaining increasing attention as a non-invasive tool for several applications such as proteomics and biomarker identification in various diseases, including cancer. The choice of the correct sampling method determines the result of the analysis. In this study, we developed and validated a robust method for tear protein quantification using ultra-high-performance liquid chromatography coupled with tandem mass spectrometry (UHPLC-MS/MS). Tear samples were collected with Schirmer strips, a low-cost and practical tool for tear collection. It is the first time that internal standards have been used to enhance the analytical performance of a method based on Schirmer strips for tear sampling, overcoming the issues widely reported in the literature regarding protein extraction and data reproducibility. Non-human proteins were used for method development, ensuring improved accuracy and analytical precision. The method demonstrated excellent recovery, high sensitivity, and reproducibility. The use of Schirmer strips, combined with this advanced analytical method, highlights their potential as a reliable support for tear protein quantification and biomarker discovery. This study provides a cost-effective and reliable workflow for tear proteome analysis and contributes to the growing field of tear-based diagnostics, making it suitable for routine clinical and research applications in precision medicine.

## 1. Introduction

The tear film plays a critical role in protecting and maintaining the health of the ocular surface by acting as the first barrier between the external environment and the eye [1]. Tear fluid is a clear, hypotonic ultrafiltrate of blood plasma, which is approximately 98% water. The remaining approximately 2% contains about 500 soluble proteins with concentrations ranging from 6 to 11 mg/mL [2], lipids, electrolytes, mucins, metabolites, hormones, desquamated epithelial cells, and foreign substances from the ambient air [3]. Proteins produced by the lacrimal glands, e.g., lysozyme and lactoferrin, and a small proportion of serum proteins from the conjunctival capillaries, e.g., albumin and transferrin, can be distinguished as the major tear proteins [4]. Protein determination has enormous potential to improve the understanding of ocular surface disease [5]. Proteomic analysis of tear fluid offers a non-invasive and highly informative approach for clinical applications. The capacity to quantify alterations in human tear fluid composition has the potential to facilitate the early detection of systemic diseases, determining novel biomarkers for these clinical conditions. Tear proteomic patterns have proven to be valuable tools for the diagnosis of severe conditions such as colon, breast, and prostate cancers, as well as glaucoma [6,7,8]. For example, albumin, lactoferrin, and lysozyme are important biomarkers in Dry Eye Disease (DED). Decreased levels of lactoferrin and lysozyme are associated with lacrimal gland dysfunction and disease severity, while increased albumin levels reflect inflammation and heightened tear permeability. These biomarkers aid in distinguishing DED subtypes, such as Sjögren’s and non-Sjögren’s, and in assessing the severity of the condition [9]. It is somewhat surprising that tears and their clinical relevance have been relatively poorly studied to date [10].

Tear samples are collected using different collection techniques. The sampling has a significant influence on the results of the analysis. It is therefore prudent to be aware of the various methods of tear collection, to assess which method is employed and to identify the anatomical collection site on the ocular surface [11]. Schirmer strips, cellulose sponges, and the capillary tube method are the most commonly used techniques [12]. The Schirmer strip is a fast and simple method for tear protein sampling. These strips are typically used to measure lacrimation in patients. Subsequent analysis of the protein content requires an extraction step. Several papers in the literature reported interesting examples of proteomic studies but mainly focused on the qualitative analysis of proteins. However, it has been demonstrated that sample processing affects both the absolute and relative concentrations of proteins if compared to direct analysis. For that reason, a standardized method for quantitative protein determination is still needed [13].

Over the last decade, several proteomic strategies have helped to identify tear proteins using various advanced techniques. Electrophoretic techniques such as two-dimensional sodium dodecyl sulfate polyacrylamide gel electrophoresis (2D-SDS PAGE), liquid chromatography coupled with mass spectrometry (LC-MS), protein microarray platform, and enzyme-linked immunosorbent assay (ELISA) are the most effective technologies for tear fluid biomarker panel analysis [14]. Tear analysis remains challenging due to the highly dynamic nature of the tear proteome and the limited sample volumes available [15]. These factors demand the use of highly sensitive and specific extraction and detection techniques. One of the most accurate approaches for protein quantification is the bottom-up strategy, which involves digesting proteins into their constituent peptides and analysing them using reversed-phase liquid chromatography (RPLC) coupled with electrospray ionization (ESI) and triple quadrupole (QQQ) mass spectrometry.

Here, we developed and validated a method for the extraction and quantification of human tear proteins collected with Schirmer test strips. Albumin, lysozyme and lactoferrin were selected based on their high abundance and established relevance in tear fluid studies. These targets also provide a range of physicochemical characteristics that allow evaluation of the method’s versatility in detecting and quantifying tear proteins.

Schirmer strips were selected for tear sample collection due to their widespread use in routine clinical practice. These strips are often discarded after clinical assessment of tear production, making them an accessible and sustainable resource for research. Their practicality and capacity to capture adequate tear volume for proteomic analysis further support their use in this study. Additionally, compared to microcapillary tubes, which can be challenging to handle and may yield inconsistent tear volumes, Schirmer strips provide a standardized and reproducible collection method [16]. Unlike ophthalmic sponges or filter paper wicks, which may retain proteins unevenly, Schirmer strips ensure efficient protein recovery, making them a reliable choice for downstream proteomic applications while maintaining patient comfort.

Protein analysis was performed using ultra-high-performance liquid chromatography (UHPLC) coupled to mass spectrometry (ESI-MS/MS) using a bottom-up proteomic approach. To enhance the analytical performance of the method, internal standards (ISs) were introduced, primarily to compensate for analyte loss during sample preparation. As already reported in the literature [17], the selected ISs were animal-derived proteins, such as bovine serum albumin and chicken lysozyme, chosen for their close similarity to the target proteins and their ability to mimic their behaviour during analytical processes. This approach was employed because using isotope-labeled ISs at the peptide level does not account for extraction and proteolytic digestion. On the other hand, using entire labeled proteins presents cost issues that are often unsustainable for routine analyses; in many cases, they are not commercially available. Non-ionic surfactants were employed for protein extraction, according to the literature and the need to ensure compatibility with mass spectrometry. Recovery, matrix effect, accuracy, and precision were assessed in the entire calibration range.

To our knowledge, this is the first time that a method for the quantitative analysis of albumin, lactoferrin, and lysozyme has been validated in Schirmer strips to meet the highest analytical requirements.

The developed protocol was also applied for protein quantification through an electrophoretic technique using the Protein 230 Kit for the 2100 Bioanalyzer Systems (Agilent, Milan, Italy). The aim was to apply this protocol to a user-friendly instrument designed for clinical practice.

## 2. Results

### 2.1. Mass Spectrometry Method Development

The MRM (multiple reaction monitoring) transitions of the targeted signature peptides were identified through the SWISS-PROT database [18], SRM Atlas [19], and the literature [17,20,21,22,23]. They were verified by injection of digested standard solutions at 10 µg/mL. MRM transitions for analysis of the signature peptides in positive ionization mode are reported in Table 1.

**Figure 1:**
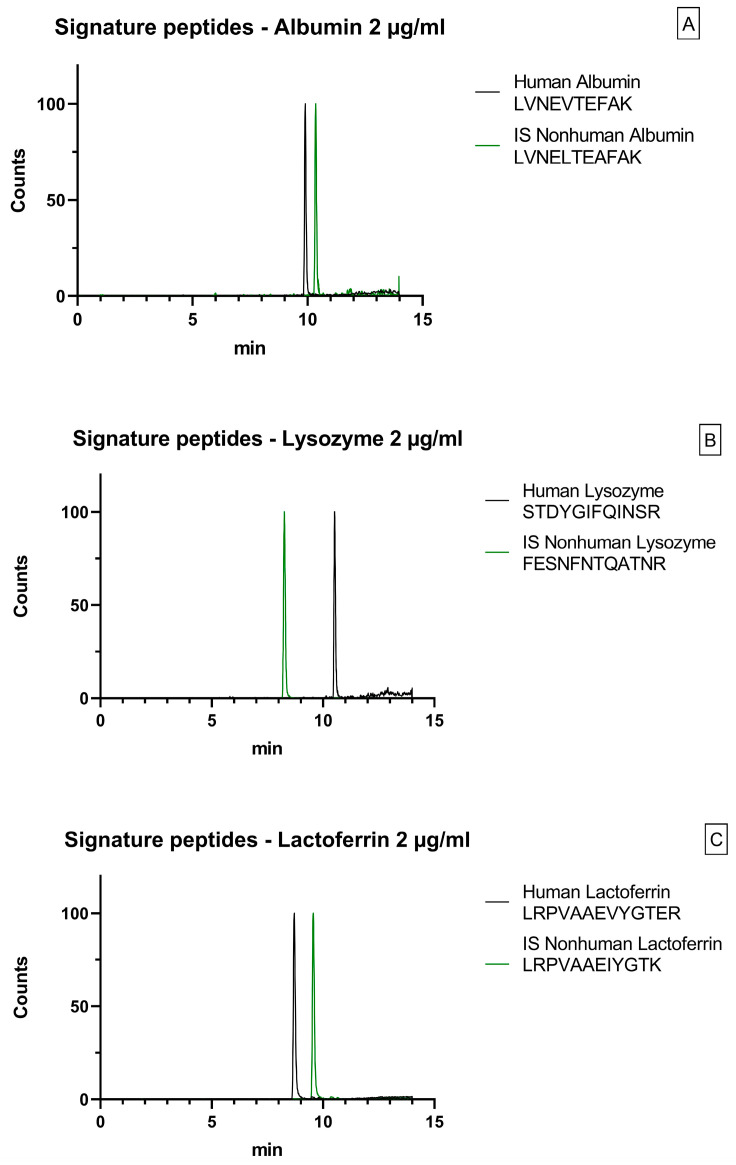
MRM signals of signature peptides for human and nonhuman proteins in 2 µg QC samples. (**A**) Albumin; (**B**) Lysozyme; (**C**) Lactoferrin.

During the development of the chromatographic method, various acidified mobile phases were tested to enhance positive ionization. Methanol and acetonitrile were used as the organic phases; however, methanol was ultimately preferred due to its superior chromatographic peak shape. Two chromatographic columns were tested: C18 BEH and Phenyl Hexyl CSH (Waters, Milford, MA, USA). The C18 column was ultimately preferred due to the reduced overlap of peptides of interest during analysis. Chromatograms are shown in Figure 1.

### 2.2. Extraction Method Development

The use of non-ionic solvents compatible with mass spectrometry was ideal for the intended application. Various extraction buffers were tested to analyse the protein content on Schirmer strips. Specifically, the following buffers were evaluated:

- Ammonium Bicarbonate (ABC) pH8
- Buffer A: 100 mM ABC pH 8 with 0.25% CHAPS
- Buffer B: 100 mM ABC pH 8 with 0.25% Triton X-100 and 0.25% CHAPS

Three quality control samples (QCs) were prepared by adding 0.2 µg (QC_low_), 2 µg (QC_medium_), and 20 µg (QC_high_) of each protein in a volume of 20 µL to each strip. Different numbers of extraction steps (n = 1, 2, 3) and extraction times (10, 20, and 30 min) were tested at 4 °C.

The Schirmer strips were cut into 5 mm sections to standardize the extraction process and optimize protein recovery. Preliminary tests indicated that this size facilitated efficient extraction while maintaining consistency across samples.

The extraction protocol was optimized by testing different buffer volumes. Increasing the extraction volume beyond 2 × 0.5 mL did not enhance protein recovery instead, it led to further dilution and a worsening of the limit of quantification. Therefore, this volume was selected as optimal for the developed method.

The cut Schirmer strips were incubated at 4 °C inside a laboratory refrigerator equipped with an orbital shaker with slow agitation (200 rpm) to facilitate protein extraction. The protocol was performed at 4 °C to minimize protein degradation and enzymatic activity. Preliminary tests conducted at room temperature showed slightly lower protein recovery and increased variability, leading to the selection of 4 °C as the optimal condition for sample processing. After incubation, the eluate was collected by centrifugation at 13,000 rpm, as previously reported by Koduri et al. [13] for a similar extraction. 

**Figure 2:**
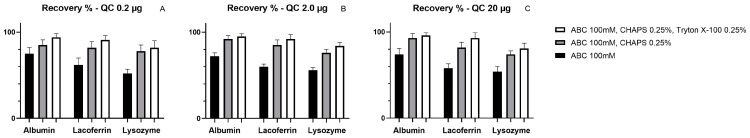
Recovery% values for the tested buffers for protein extraction in QC samples: Ammonium bicarbonate; ABC 100 mM, 0.25% CHAPS X-100; ABC 100 mM with 0.25% Triton X-100 and 0.25% CHAPS. Figure legend: Panels (**A**–**C**) correspond to the three tested protein deposition amounts: 0.2, 2, and 20 µg, respectively.

Using Buffer A (100mM ABC, 0.25% CHAPS X-100), the following results were obtained for QC_medium_ 2 µg: lysozyme 76% ± 4%, albumin 92% ± 4% and lactoferrin 85% ± 6%. The buffer with both CHAPS and Triton (Buffer B) achieved better recovery percentages, especially for albumin and lactoferrin (Rec% respectively of 95% ± 3% and 92% ± 5%), while the recovery for lysozyme was lower (84% ± 4%). Data are reported in Figure 2. The results showed that Buffer B was the most effective for protein extraction. No significant differences (*p* > 0.05) were found with additional extraction steps or longer extraction times. 

### 2.3. Method Validation Results

A linearity range was determined for each protein from 0.01 µg/mL to 100 µg/mL. The presence of interferents was excluded in each MRM spectrum using the extract of a blank Schirmer strip, applying the protocol described in Section 4.4.

The presence of a negligible matrix effect (ME) was determined, as described in Section 4.5, with ME% values from 1.3% to 2.5% for all proteins (Supplementary Table S1). Accuracy and precision were determined as reported in Section 4.5 with bias < 5% and CV < 5% for each protein, as reported in the Supplementary Materials (Table S2). Limit of detection (LOD) and limit of quantification (LOQ) for albumin, lactoferrin and lysozyme were respectively 1–10 ng/mL, 0.1–1 ng/mL, and 0.5–5 ng/mL.

### 2.4. Application in Real Tear Samples

**Figure 3:**
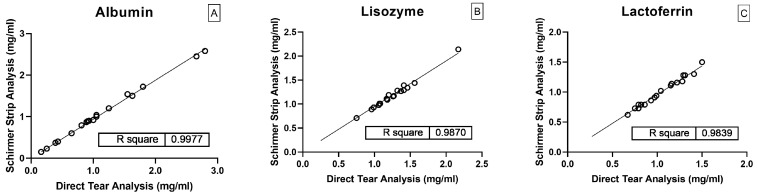
Direct tear analysis versus Schirmer strip extract analysis related to. (**A**) Albumin; (**B**) Lysozyme; (**C**) Lactoferrin.

Twenty real tear samples were split and analyzed either directly by mass spectrometry, or after being adsorbed onto a Schirmer strip and extracted as reported above. Figure 3 reports results for each quantified protein, comparing the outcomes obtained from direct tear analysis with those from analysis of the Schirmer strip extracts.

Error expressed as bias percentage was calculated by comparing the direct analysis method with the developed method involving Schirmer strip extraction followed by mass spectrometry analysis. Bias% was lower than 8%. The results demonstrate excellent recovery of protein content through the developed extraction protocol. Additionally, the proper use of the ISs effectively corrected for variability in the extraction process and digestion step. An ANOVA test was performed to compare the data obtained from the two analytical approaches. The analysis showed no statistically significant differences between the methods (*p*-value > 0.05). This result indicates the variability observed between the two methods is not statistically significant and the methods can be considered comparable in performance.

A separate set of ten tear samples was used to evaluate the performance of the developed extraction protocol without the use of ISs. These samples were split and analysed directly or after being adsorbed onto a Schirmer strip via 2100 Bioanalyzer Systems (Agilent, Milan, Italy), compatible with the used extraction buffer but without the presence of the IS. An example of the output of the Bioanalyzer Systems is reported in the Supplementary Materials (Figures S1 and S2). The bias percentages were calculated with a range between 7% and 15% for albumin and lactoferrin (Supplementary Material Table S3). Higher bias% values were obtained for lysozyme (25–30%). This is a reasonable result considering the absence of an applicable IS for this technique. It highlights the need to improve the electrophoresis method with the use of an appropriate IS to increase analytical performance, as was used in the mass spectrometry-based method developed in this study.

## 3. Discussion

In this study, we developed and validated a novel method for the accurate quantification of tear proteins collected with Schirmer strips, utilizing non-human proteins as ISs.

The gold standard for protein quantification typically involves the use of isotopically labeled ISs. However, introducing isotopically labeled tryptic peptides post-extraction does not compensate for protein losses that occur during the initial handling and processing steps. Alternative strategies, such as producing labelled proteins through techniques like SILAC (Stable Isotope Labelling by Amino Acids in Cell Culture) or ICAT (Isotope-Coded Affinity Tags) or purchasing pre-labeled proteins, could offer more comprehensive corrections for protein losses. However, these methods are associated with significant costs and logistical challenges, limiting their applicability in routine clinical workflows.

Using a non-human protein as an IS emerged as a practical and cost-effective alternative [17]. This approach proved suitable for our objective, allowing us to minimize potential variations during sample preparation and correct for potential losses of intact proteins, leading to more consistent data and improved analytical precision. Despite its success, this method requires careful evaluation and validation for each target protein, as addressed in the present study.

To the best of our knowledge, this is the first time such a methodology has been applied to Schirmer strips. Using Schirmer strips for tear collection proved to be an efficient and practical approach, offering a simple, non-invasive method for obtaining samples suitable for protein analysis. Beyond its novelty, this study addresses the growing need to standardize methods for tear proteome analysis—an issue that remains a significant point of discussion within the scientific community. Our findings demonstrate the potential of Schirmer strips as a reliable tool for accurately determining tear protein content. This study lays the foundation for future applications of this method in routine clinical and research settings, such as biomarker discovery, emphasizing the need for ongoing optimization and validation to extend its scope in precision medicine. This method serves as a proof of concept, initially applied to these proteins but designed to be extended to other proteins or different molecular targets of interest. The use of appropriate ISs directly on Schirmer strips is crucial to reaching high analytical standards, as it accounts for the variability introduced by this collection support method.

This study presents a novel method for protein quantification in tear samples using Schirmer strips and mass spectrometry. However, some limitations must be acknowledged. The method requires protein-specific validation for each target to ensure compatibility between the extraction process and the chosen IS. Such validation is essential to confirm that the IS effectively corrects for variability during the extraction and digestion steps. While the method showed excellent recovery for the selected proteins, it has not yet been tested on the full range of proteins present in tear fluid. Moreover, the efficiency of the extraction process may vary among proteins, particularly those with extreme physicochemical properties, such as highly hydrophobic or low-abundance proteins.

## 4. Materials and Methods

### 4.1. Reagent and Standard Solution

Water of HPLC-MS grade (Millipore, Merck, Darmstadt, Germany) was produced using a Milli-Q Synthesis A 10 depurative system (Merck, Molsheim, France). Ammonium bicarbonate (>99.0%) and formic acid were purchased from Sigma-Aldrich (Saint Louis, MO, USA). LC-MS-grade methanol and acetonitrile, human albumin P02768 (Alb), lysozyme P61626 (Lys) and lactoferrin P02788 (Lact) were purchased from Merck (Germany). The analytical standards for human albumin, lysozyme, and lactoferrin were prepared by dissolving them in phosphate-buffered saline (PBS) to achieve a concentration of 5 mg/mL. Bovine serum albumin P02769 (BSA), Lysozyme from chicken egg white P00698 and Lactoferrin from bovine milk P24627 were purchased from Merck and used as ISs, prepared according to the previously described standards. Triton X-100 and Chaps (laboratory grade) were purchased from Sigma-Aldrich.

### 4.2. Targeted Mass Spectrometry Method for Protein Quantification

A UHPLC-MS/MS method was developed and validated to analyze albumin, lactoferrin, lysozyme, and ISs. Tear and standard samples were prepared using the Thermo Scientific Pierce Trypsin Protease Kit for bottom-up mass spectrometry analysis. This kit facilitated protein enzymatic digestion into signature peptides suitable for subsequent mass spectrometry analysis. An amount of 20 µL of standard or sample was required for the digestion step and mass spectrometry analysis.

Liquid chromatography was performed using a UHPLC 1290 Infinity II ultra-high-performance liquid chromatograph (Agilent Technologies, Inc., Andover, MA, USA) coupled to a 6495 LC/TQ triple quadrupole mass spectrometer (Agilent Technologies, Inc.), equipped with an ESI source, operating in multiple reaction monitoring (MRM) acquisition mode.

Tryptic peptides were separated using a C18 analytical column (ACQUITY UPLC BEH, 150 mm × 2.1 mm, 1.7 μm particle size). The mobile phases consisted of solvent A (0.1% formic acid in water) and solvent B (0.1% formic acid in methanol) in gradient elution mode: B 5%, B 25% until 5 min, B 30% until 7 min, B 80% until 11 min and held until 14 min (flux 0.3 mL/min). Equilibration time was 3 min and total runtime was 17 min.

Data processing was performed using MassHunter software (Agilent Technologies, Inc., Andover, MA, USA). Statistical analysis and graph generation were carried out using Prism version 10.4.1 (GraphPad Software, San Diego, CA, USA). The graphical abstract was created with BioRender (BioRender, Toronto, ON, Canada).

### 4.3. Quality Control Samples and Real Samples

QC Schirmer strips were prepared by adding known quantities of protein at three levels: 0.2 µg, 2 µg, and 20 µg (low, medium, and high QCs). Fixed amount of the IS mix (2 µg of each IS, volume of 10 µL) was added to each QC and real sample.

Tear samples were obtained anonymously from 20 volunteers at the Sant’Orsola-Malpighi Polyclinic and made available by the Ocular Surface Analysis and Translational Research Laboratory at the University of Bologna. The samples were collected in the morning to avoid variations in protein content due to circadian rhythms. Tears were collected from both eyes of each subject using a micropipette. Two microliters were used for direct analysis via mass spectrometry. IS and the remainder of the tear sample were deposited onto a Schirmer strip, extracted, and analysed by mass spectrometry.

### 4.4. Extraction Method for Schirmer Strips

**Figure 4:**
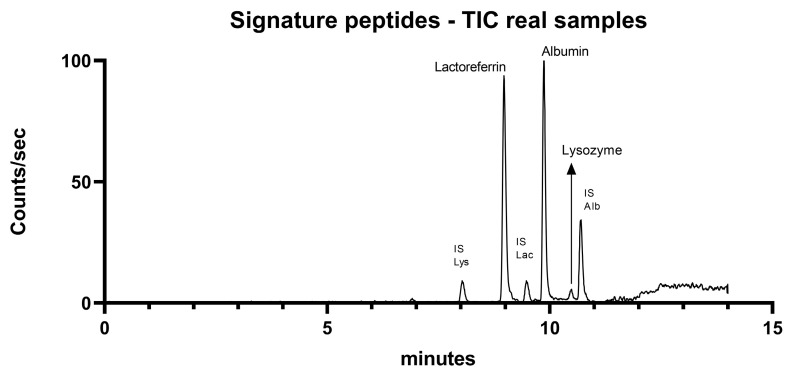
Total Ion Current (TIC) chromatogram of a representative tear sample adsorbed on a Schirmer strip, analyzed using the optimized UHPLC-MS/MS method.

The protein content of QCs and real samples was extracted in 100 mM ABC with 0.25% Triton X-100 and 0.25% CHAPS. The choice to use non-ionic surfactants was based on the literature [13] and the necessity to develop an extraction method compatible with mass spectrometry. Schirmer strips (5 mm) were incubated in 500 µL of extraction buffer at 4 °C with agitation (200 rpm) for 30 min. Extraction was performed twice to optimize protein recovery while minimizing dilution. Eluate was collected by centrifugation at 13,000 rpm, following Koduri et al. [13]. Figure 4 illustrates the chromatographic profile of the protein content extracted from Schirmer strips using the developed mass spectrometry method.

In this study, the exact amount of protein deposited on the QC strips was known, as well as the exact tear volume for the real samples. On the other hand, in normal practice, there is a necessity to determine the sample volume deposited on the strip. A conversion factor was determined to convert the length of the wetted portion of the strip into the volume of tear adsorbed by the strip during a Schirmer test. This approach has been previously reported in the literature [13], although it is necessary to account for potential variations depending on the type of strip used. The conversion factor was accurately determined in triplicate using six different standard solution volumes of the investigated proteins (5, 10, 15, 20, 25, 30, 35, 40 µL) at 1 mg/mL. The ratio between millimeters and tear volume was 1.15 mm/µL (SD ± 0.05).

### 4.5. Method Validation

The method was validated using standard samples, QCs, and real samples. Protein content was determined using mass spectrometry after the digestion step as described above.

Recovery percentage (Rec %) was determined as the ratio between the amount of each extracted protein from QCs and spiked extraction buffers with the same protein content present on the strip. A clean strip was immersed in the buffer before extraction to exclude differences in ME.

ME% was determined at three concentration levels for each protein. ME was determined as the ratio between the protein response in spiked extraction buffers and the protein response in net standard solutions.

A seven-point calibration curve (0.1, 0.5, 1, 5, 10, 50, and 100 µg/mL), obtained by dilution of the standard solution with PBS, was used for the linearity study and quantification of the different proteins. The IS mix was added prior to digestion with a fixed amount of 2 µg/mL.

Accuracy and precision were determined in triplicate using QCs, using the IS calibration curve. Accuracy was assessed by comparing the differences between the expected protein content and the extracted protein content after Schirmer strip extraction. The Bias% was determined for each QC using the formula:

Bias% = ((extracted protein content − expected protein content)/expected protein content) × 100(1)

The precision was determined for QCs in triplicate, calculating the percent coefficient of variation (CV%):

CV% = (SD/Mean) × 100(2)

where SD is the standard deviation and Mean is the average value of each extracted protein.

The LOD and LOQ of the method for each single analyte were experimentally determined by injecting step-wise dilution of the standard solution after the digestion step.

### 4.6. Electrophoresis for Protein Quantification

Simultaneously, the Protein 230 Kit for 2100 Bioanalyzer Systems (Agilent, Milan, Italy) was used for protein quantification in real extracted samples through capillary electrophoresis, testing the compatibility of the developed extraction buffer with this tool for clinical use.

Unlike mass spectrometry, capillary electrophoresis cannot differentiate between human and nonhuman proteins. In accordance with this, real tear samples (n = 10) were split into two aliquots. The first aliquot of 2 µL was analysed directly via capillary electrophoresis, while the remaining sample was adsorbed onto Schirmer strips without the addition of ISs and extracted and analysed with the 2100 Bioanalyzer system (Agilent).

## Figures and Tables

**Table 1:** MRM parameters of the precursor ion, product ion, collision energy, and retention time for each signature peptide and IS.

Protein	Signature Peptide	Precursor Ion	Product Ion	Collision Energy	Retention Time (min)
Albumin	LVNEVTEFAK	575.3	595.3 [y5] *; 694.4 [y6]	25; 20	9.85
IS Albumin	LVNELTEAFAK	582.3	708.4 [y6] *; 837.4 [y7]	25;20	10.28
Lactoferrin	LRPVAAEVYGTER	487.6	462.2 [y4] *; 737.4 [b7]	20;20	8.80
IS Lactoferrin	LRPVAAEIYGTK	659.3	737.4 [b7] *; 850.5 [b8]	20;20	9.52
Lysozyme	STDYGIFQINSR	700.8	764.4 [y6] *; 304.1 [b3]	25;25	10.11
IS Lysozyme	FESNFNTQATNR	714.8	1152.5 [y10] *; 804.3 [y7]	25;25	8.18

* Indicates the ion for quantitative analysis.

## Data Availability

The raw data will be made available by the corresponding author on reasonable request.

## Supplementary Materials

The supporting information can be downloaded at: https://www.mdpi.com/article/10.3390/ijms26052041/s1.

## Abbreviations

The following abbreviations are used in this manuscript:

2D-SDS PAGE	two-dimensional sodium dodecyl sulfate polyacrylamide gel electrophoresis
LC-MS	liquid chromatography coupled with mass spectrometry
ELISA	enzyme-linked immunosorbent assay
RPLC	reversed-phase liquid chromatography
ESI	electrospray ionization
QQQ	triple quadrupole
UHPLC	ultra-high-performance liquid chromatography
IS	internal standard
QCs	quality control
MRM	multiple reaction monitoring
LOD	limit of detection
LOQ	limit of quantification
Alb	human albumin
Lys	human lysozyme
Lact	human lactoferrin
