# Supplementary material for: Tear Sampling and Biomarker Discovery: A Robust Workflow for Routine Clinical Applications Using UHPLC-MS/MS and Schirmer Strips
Source: Int J Mol Sci. 2025 Feb 26;26(5):2041. doi: 10.3390/ijms26052041 (PMC11900304; doi:10.3390/ijms26052041)

Table S1. Matrix effect value for albumin, lysozyme and lactoferrin at three different concentration levels (n=3).

**Matrix effect (Me%)**			
**Protein**	**0.2 µg**	**2 µg**	**20 µg**
**Albumin**	2.90 ± 0.29	3.20 ± 0.32	3.50 ± 0.35
**Lactoferrin**	1.80 ± 0.18	2.00 ± 0.20	2.30 ± 0.23
**Lysozyme**	2.10 ± 0.21	2.30 ± 0.23	2.60 ± 0.26

Table S2. Accuracy and precision value for albumin, lysozyme and lactoferrin for QC samples (n=3).

**Protein**	**QC Low (2 µg)**		**QC Medium (2 µg)**		**QC High (20 µg)**	
	**Bias %**	**CV %**	**Bias %**	**CV %**	**Bias %**	**CV %**
**Albumin**	2.30 ± 0.09	3.91%	2.60 ± 0.12	4.62%	3.10 ± 0.14	4.52%
**Lactoferrin**	1.90 ± 0.08	4.21%	2.20 ± 0.09	4.09%	2.60 ± 0.11	4.23%
**Lysozyme**	2.00 ± 0.07	3.50%	2.30 ± 0.10	4.35%	2.80 ± 0.11	3.93%

Table S3. Results for albumin, lysozyme and lactoferrin determined using electrophoresis in 10 tear samples.

Albumin (mg/ml)		Lactoferrin (mg/ml)		Lysozyme (mg/ml)	
Direct analysis	After Shirmer extraction	Direct analysis	After Shirmer extraction	Direct analysis	After Shirmer extraction
1.12	0.98	1.10	1.00	1.12	0.86
0.73	0.65	1.28	1.11	0.98	0.74
1.27	1.13	1.45	1.29	1.15	0.89
0.61	0.53	0.98	0.87	1.23	0.92
1.35	1.18	1.35	1.20	1.08	0.83
0.92	0.8	1.22	1.08	1.25	0.95
1.45	1.3	1.56	1.41	0.92	0.71
0.49	0.44	1.10	0.98	1.10	0.80
1.08	0.95	1.25	1.13	0.87	0.67
0.15	0.08	1.08	0.97	1.09	0.84

Figure S1. Representation of electropherograms obtained with the Agilent 2100 Bioanalyzer system (20).

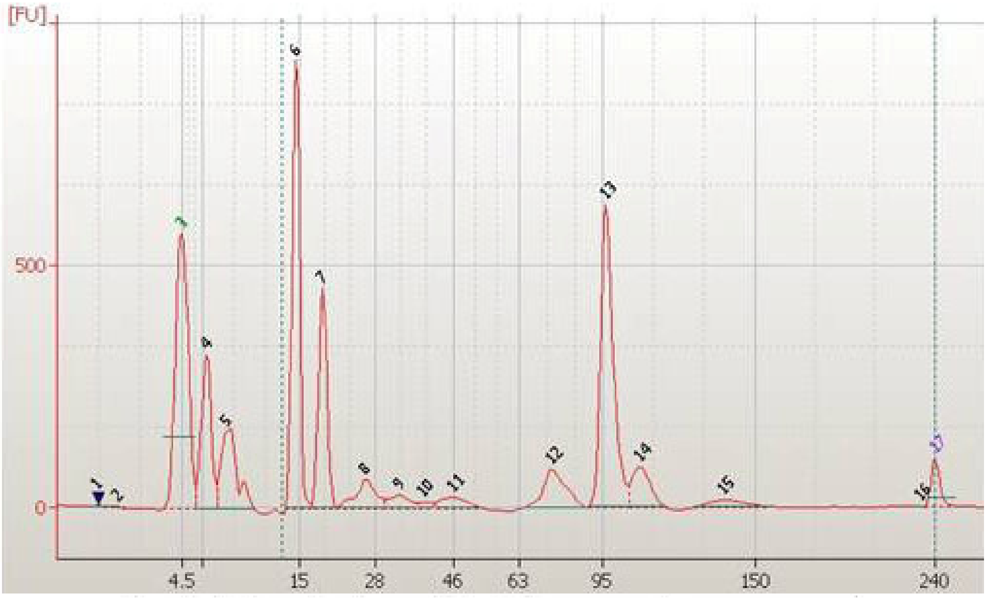

Figure S2. Electrophoresis gel output from the Agilent 2100 Bioanalyzer system, showing the separation of tear proteins based on molecular weight.

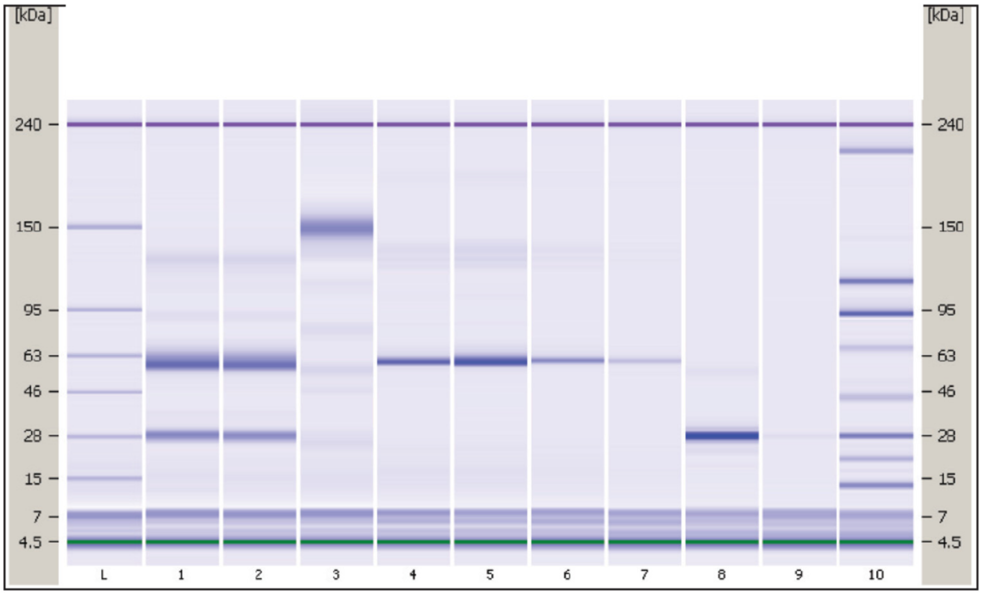

Supplement: Supplementary file 1 [file ijms-26-02041-s001.zip › ijms-3446890-supplementary.pdf]
